# Supplementary material for: A pediatric brain tumor atlas of genes deregulated by somatic genomic rearrangement
Source: Nat Commun. 2021 Feb 10;12:937. doi: 10.1038/s41467-021-21081-y (PMC7876141; doi:10.1038/s41467-021-21081-y)
Supplement: Supplementary file 1 — Supplementary Information [file 41467_2021_21081_MOESM1_ESM.pdf]

Supplementary Information

Zhang et al. “A pediatric brain tumor atlas of genes deregulated by somatic genomic rearrangement”

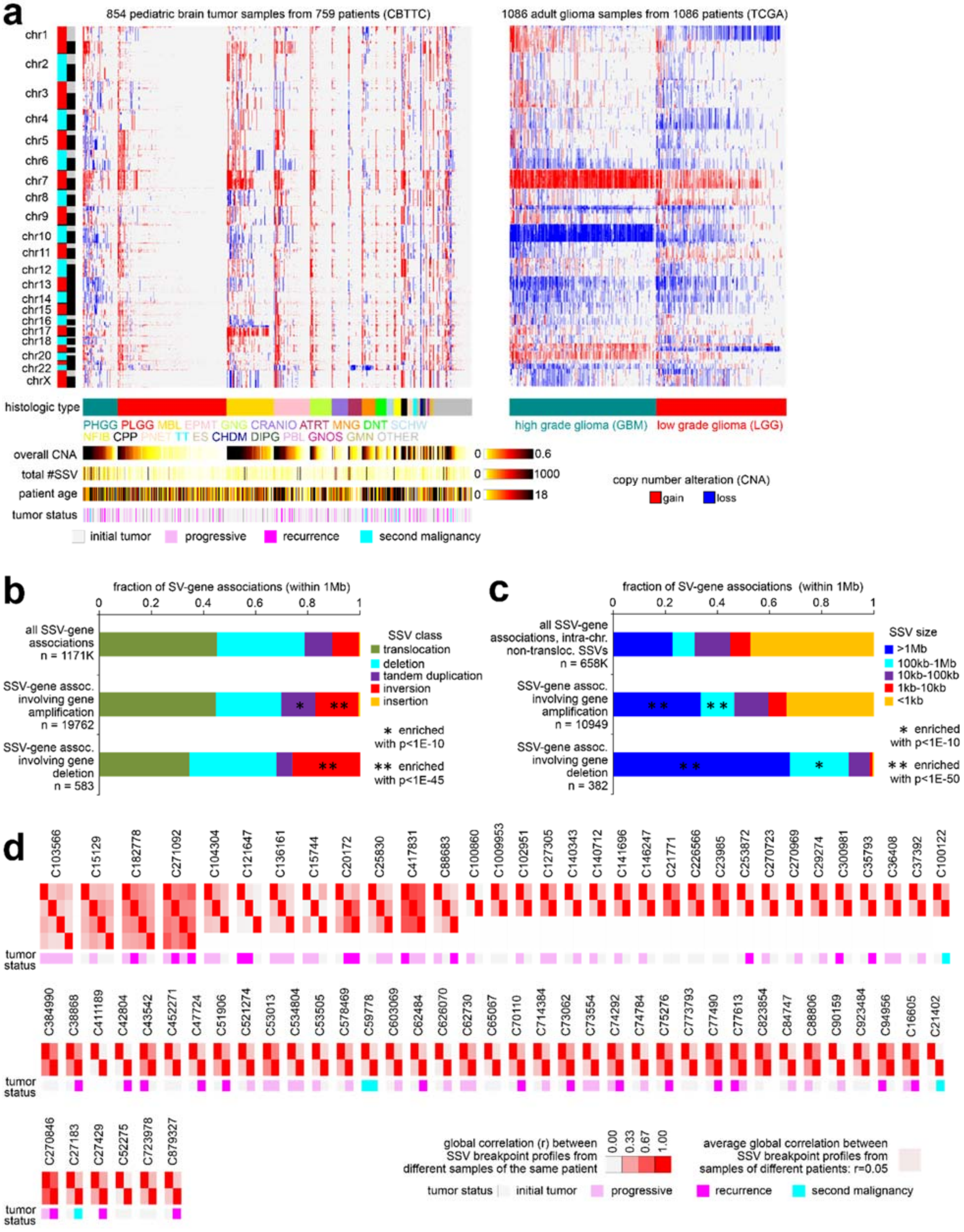
a
854 pediatric brain tumor samples from 759 patients (CBTTC)
1086 adult glioma samples from 1086 patients (TCGA)
chr1
chr2
chr3
chr4
chr5
chr6
chr7
chr8
chr9
chr10
chr11
chr12
chr13
chr14
chr15
chr16
chr17
chr18
chr20
chr22
chrX
histologic type
PHGG PLGG MBL EPMT GNG CRANIO ATRT MNG DNT SCHW
NFIB CPP PNET TT ES CHDM DIPG PBL GNOS GMN OTHER
high grade glioma (GBM)
low grade glioma (LGG)
overall CNA
0
0.6
total #SSV
0
1000
patient age
0
18
tumor status
initial tumor
progressive
recurrence
second malignancy
copy number alteration (CNA)
gain
loss
b
fraction of SV-gene associations (within 1Mb)
0 0.2 0.4 0.6 0.8 1
all SSV-gene associations n = 1171K
SSV-gene assoc. involving gene amplification n = 19762
SSV-gene assoc. involving gene deletion n = 583
SSV class
translocation
deletion
tandem duplication
inversion
insertion
* enriched with p<1E-10
** enriched with p<1E-45
c
fraction of SV-gene associations (within 1Mb)
0 0.2 0.4 0.6 0.8 1
all SSV-gene associations, intra-chr. non-transloc. SSVs n = 658K
SSV-gene assoc. involving gene amplification n = 10949
SSV-gene assoc. involving gene deletion n = 382
SSV size
>1Mb
100kb-1Mb
10kb-100kb
1kb-10kb
<1kb
* enriched with p<1E-10
** enriched with p<1E-50
d
C103566 C15129 C182778 C271092 C104304 C121647 C136161 C15744 C20172 C25830 C417831 C88683 C100860 C1009953 C102951 C127305 C140343 C140712 C141696 C146247 C21771 C226566 C23985 C253872 C270723 C270969 C29274 C300981 C35793 C36408 C37392 C100122
tumor status
C384990 C38868 C411189 C42804 C43542 C452271 C47724 C51906 C521274 C53013 C534804 C53505 C578469 C59778 C603069 C62484 C626070 C62730 C65067 C70110 C714384 C73062 C73554 C74292 C74784 C75276 C773793 C77490 C77613 C823854 C84747 C88806 C90159 C923484 C94956 C16605 C21402
tumor status
C270846 C27183 C27429 C52275 C723978 C879327
tumor status
global correlation (r) between SSV breakpoint profiles from different samples of the same patient
0.00 0.33 0.67 1.00
average global correlation between SSV breakpoint profiles from samples of different patients: r=0.05
tumor status
initial tumor
progressive
recurrence
second malignancy

Supplementary Figure 1, related to Figure 1. Additional information regarding global SSV and CNA patterns across pediatric brain tumors. (a) Across the 854 pediatric brain tumor tumors from CBTTC (left) and the 1086 adult glioma samples from TCGA (right), cytoband-level CNA patterns are represented (red, copy gain; blue, copy loss). Overall CNA for each sample profile is the standard deviation of the gene-level CNA values collapsed into cytoband regions. Genome doubling events are not represented here. **(b-c)** For SSV-gene associations (each association involving an SSV breakpoint occurring within 1Mb of gene start site), breakdown by SSV class (part b) and SSV size (part c) is provided for all associations, associations involving high-level gene amplification, and associations involving deep gene deletions. SSV size chart involves SSVs that are intra-chromosomal and which are not translocations. P-values for enrichment (as compared to the entire set of associations) by chi-squared test. SSVs associated with gene amplification or deletion are found here to be highly enriched for inversion SSVs, as well as for SSVs >100kb in size. **(d)** Evidence of inter-tumoral heterogeneity within patients by global analysis of SSV breakpoint patterns surveyed across multiple tumor samples from the same patient. A data matrix of absolute relative distances for 18,786 genes and 854 CBTTC samples was assembled, with a relative distance metric of 1Mb being applied for any tumor with no breakpoints within 1Mb of the gene. Using this breakpoint pattern matrix (with relative distance values being log-transformed, the global correlation in SSV breakpoint profiles was taken between CBTTC tumors taken from the same patients. Inter-profile correlations involving 170 tumors from 75 patients are represented. Tumor status for each sample (initial tumor, progressive, recurrence, second malignancy) is also indicated. While in many instances, the global correlation between tumors from the same patients is observed to be quite high, these correlation r-values are much less than 1, and in many other instances, the level of correlation is close to that of unrelated tumors from different patients.

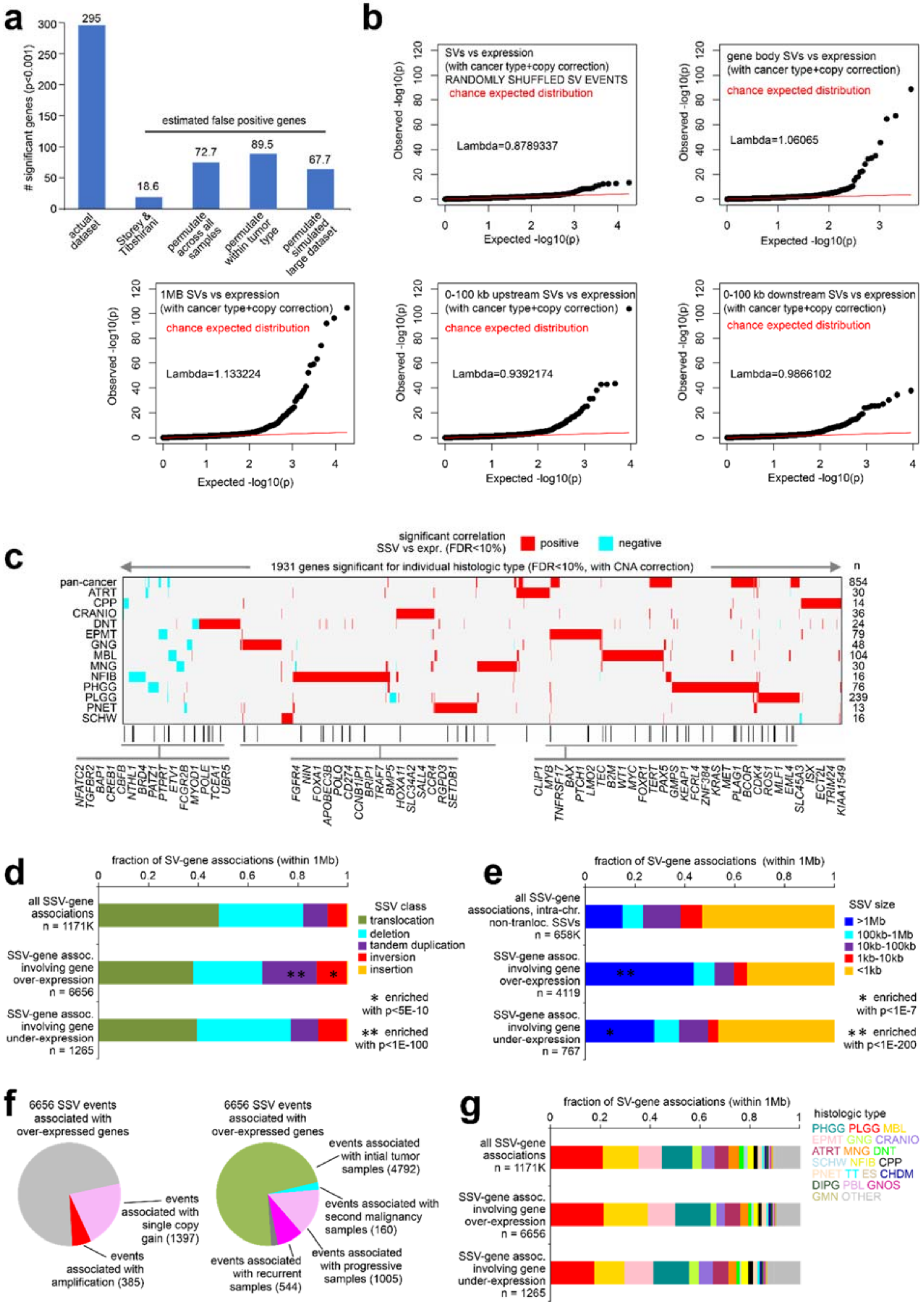
a
significant genes (p<0.001)
295
estimated false positive genes
18.6
72.7
89.5
67.7
actual dataset
Storey & Tibshirani
permutate across all samples
permutate within tumor type
permutate simulated large dataset
b
SVs vs expression
(with cancer type+copy correction)
RANDOMLY SHUFFLED SV EVENTS
chance expected distribution
Lambda=0.8789337
gene body SVs vs expression
(with cancer type+copy correction)
chance expected distribution
Lambda=1.06065
1MB SVs vs expression
(with cancer type+copy correction)
chance expected distribution
Lambda=1.133224
0-100 kb upstream SVs vs expression
(with cancer type+copy correction)
chance expected distribution
Lambda=0.9392174
0-100 kb downstream SVs vs expression
(with cancer type+copy correction)
chance expected distribution
Lambda=0.9866102
Observed -log10(p)
Expected -log10(p)
c
significant correlation SSV vs expr. (FDR<10%)
positive
negative
1931 genes significant for individual histologic type (FDR<10%, with CNA correction)
n
pan-cancer 854
ATRT 30
CPP 14
CRANIO 36
DNT 24
EPMT 79
GNG 48
MBL 104
MNG 30
NFIB 16
PHGG 76
PLGG 239
PNET 13
SCHW 16
NFATC2 TGFBR2 BAP1 CREB1 CBFB NTHL1 BRD4 PATZ1 PTPRT ETV1 FCGR2B MYOD1 POLE TCEA1 UBR5
FGFR4 NIN FOXA1 APOBEC3B POLQ CD274 CCNB1IP1 BRIP1 TRAF7 BMP5 HOXA11 SLC34A2 SALL4 CCR4 RGPD3 SETDB1
CLIP1 MYB TNFRSF17 BAX PTCH1 LMO2 TEC B2M WT1 MYC FOXR1 TERT PAX5 GMPS KEAP1 FCRL4 ZNF384 KRAS MET PLAG1 BCOR CDK4 ROS1 MLF1 EML4 SLC45A3 ISX ECT2L TRIM24 KIAA1549
d
fraction of SV-gene associations (within 1Mb)
all SSV-gene associations n = 1171K
SSV-gene assoc. involving gene over-expression n = 6656
SSV-gene assoc. involving gene under-expression n = 1265
SSV class
translocation
deletion
tandem duplication
inversion
insertion
* enriched with p<5E-10
** enriched with p<1E-100
e
fraction of SV-gene associations (within 1Mb)
all SSV-gene associations, intra-chr. non-tranloc. SSVs n = 658K
SSV-gene assoc. involving gene over-expression n = 4119
SSV-gene assoc. involving gene under-expression n = 767
SSV size
>1Mb
100kb-1Mb
10kb-100kb
1kb-10kb
<1kb
* enriched with p<1E-7
** enriched with p<1E-200
f
6656 SSV events associated with over-expressed genes
events associated with single copy gain (1397)
events associated with amplification (385)
6656 SSV events associated with over-expressed genes
events associated with intial tumor samples (4792)
events associated with second malignancy samples (160)
events associated with progressive samples (1005)
events associated with recurrent samples (544)
g
fraction of SV-gene associations (within 1Mb)
all SSV-gene associations n = 1171K
SSV-gene assoc. involving gene over-expression n = 6656
SSV-gene assoc. involving gene under-expression n = 1265
histologic type
PHGG PLGG MBL
EPMT GNG CRANIO
ATRT MNG DNT
SCHW NFIB CPP
PNET TT ES CHDM
DIPG PBL GNOS
GMN OTHER

Supplementary Figure 2, related to Figure 2. Additional information regarding altered gene expression patterns associated with nearby SSV breakpoints. (a) Estimated false discovery rates (FDRs) involving the top significant genes with SSV-expression associations ($p<0.001$, distance metric method, correcting for both tumor type and CNA). FDRs are estimated using the following methods: 1) Storey and Tibshirani method[1], 2) permutation of SSV profiles across all 854 samples with respect to the expression profiles, 3) permutation of SSV profiles with respect to the expression profiles but shuffling within tumor type (given the strong expression versus tumor type associations), 4) permutation of SSV profiles with respect to the expression profiles but shuffling within tumor type and using a large simulated dataset. All permutation testing results are based on 1000 permutations. For the large simulated dataset, five copies of the actual 854-tumor matrices were concatenated together to make one large dataset of 4270 samples, where the same relationships as represented in the 854-tumor dataset are present, but with the larger sample size allowing for more permutations that have little or no overlap with the actual dataset (being closer to a truly random distribution). As expected, the large simulated dataset shows fewer estimated false positive genes as compared to permutation results using the original 854-tumor dataset, where a larger sample size affords more power. Permuting across all tumor profiles using the large simulated dataset (result not plotted) indicates that ~43 of the 295 $p<0.001$ genes might be false positives by this estimate. **(b)** QQ plots of the linear regression p-values from the SSV events versus expression correlations (correcting for both tumor type and CNA), for SSV breakpoints falling within each of the indicated regions surrounding the gene. The top left QQ plot represents results from the 0-20kb upstream SV events being randomly permuted relative to the expression profiles. Lamda values represent [median of p-values]/0.456, intended as a measure of the observed versus expected median (the concept being borrowed from Genome-Wide Association Studies and applied here to the differential expression analyses). **(c)** Heat map of differential t-statistics by tumor type, evaluating gene expression alterations with nearby SSV breakpoint (red, positive correlation with breakpoint; white, not significant with FDR>10%), for 1931 genes significant for one or more individual tumor types (FDR<10% by distance metric method, correcting for CNA). Genes listed by name are cancer related by COSMIC. **(d)** Breakdown by SSV class, for all SSVs-gene associations involving a breakpoint within 1Mb of gene start in the dataset, for the SSVs-gene associations involving with gene over-expression (using FDR < 10% by distance metric method, with corrections for tumor type and CNA, and expression>0.4SD from sample median), and for the SSVs-gene associations involving with gene under-expression (using FDR < 10% by distance metric method, with corrections for tumor type

and CNA, and expression<-0.4SD from sample median). P-values for enrichment by chi-squared test. **(e)** Similar to part c, but with breakdown by SSV size, and involving just those SSVs that are intra-chromosomal and which are not translocations. P-values for enrichment by chi-squared test. **(f)** For 6350 events of an SSV breakpoint being associated with over-expression of a nearby gene (FDR<10% by distance metric method, with corrections for tumor type and CNA, and expression>0.4SD from median for the case harboring the breakpoint), breakdowns are provided according to events involving gain or high-level amplification of the same gene (defined using threshold CNA calls), and according to events by the tumor status (initial tumor, progression, recurrent, or second malignancy). **(g)** Similar to part c, but with breakdown by tumor type.

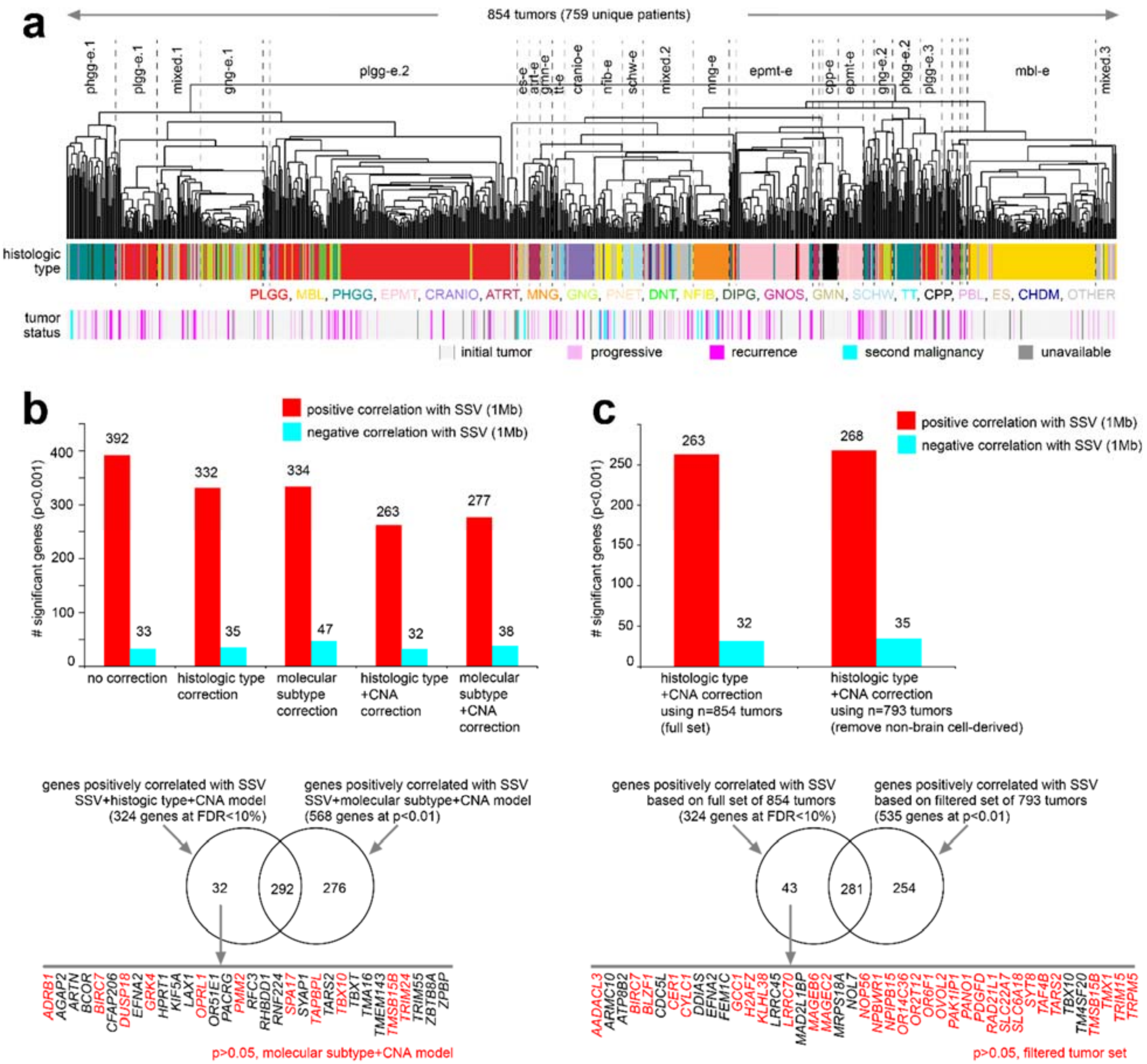

Supplementary Figure 3, related to Figure 2. Use of molecular subtype instead of histologic type to define SSV-associated altered expression. (a) Hierarchical clustering of the top 2000 most variable genes carried out on the CBTTC RNA-seq data. As expected, the tumors broadly segregated according to histologic type, although there is some variability in terms of the grouping of tumors of a given histology. From these results, we could broadly classify the CBTTC tumors according to a molecular subtype. Here we use a designation of "-e" for "enriched," in order to make a distinction from the histology-based type. For example "phgg-e.1" represents a "phgg-enriched" cluster (with two such clusters identified, hence these are distinguished by "1" versus "2"). These molecular subtype designations are provided in the sample-level information table in Data File S1. **(b)** Numbers of significant genes (p<0.001), showing a correlation between

expression and SSVs occurring within 1Mb of the gene (using distance metric method). Different linear regression models are considered here, as indicated, including using molecular-based subtype (from part a) instead of the histologic designation. Venn diagram below represents the overlap between the genes positively correlated (FDR<10%) with SSV breakpoint when using histologic type and the genes positively correlated (p<0.01) with SSV breakpoint when using molecular subtype. Most of the 324 genes significant at FDR<10% using the histologic type+CNA model (from main Figure 2b), were also significant in the alternate molecular subtype+CNA model, with just 11 genes not being significant (p>0.05) for the model using molecular subtype. **(c)** Some tumor types represented in CBTTC—including Ewing's sarcoma (ES, n=6), germinoma (GMN, n=4), Langerhans cell histiocytosis (LCH, n=4), malignant peripheral nerve sheath tumor (MPNST, n=3), and neuroblastoma (NBL, n=2)—originate from cell types not specific to the brain, even if the CBTTC tumors were obtained from the brain region. Removing the above tumors in question, along with metastatic secondary tumors and Not Otherwise Specified or “other” tumors, we are left with a set of 793 tumors out of the full set of 854. Numbers of significant genes (p<0.001), showing a correlation between expression and SSVs, using the full 854 tumor cohort versus the 793 cohort, are shown. Venn diagram below represents the overlap between the genes positively correlated with SSVs when using the 854-tumor set versus the 793-tumor subset. Most of the 324 genes significant at FDR<10% using the histologic type+CNA model and the full 854-tumor set (from main Figure 2b), were also significant in the alternate analysis using the 793 tumor subset, with just 31 genes not being significant (p>0.05) for the 793 tumor subset.

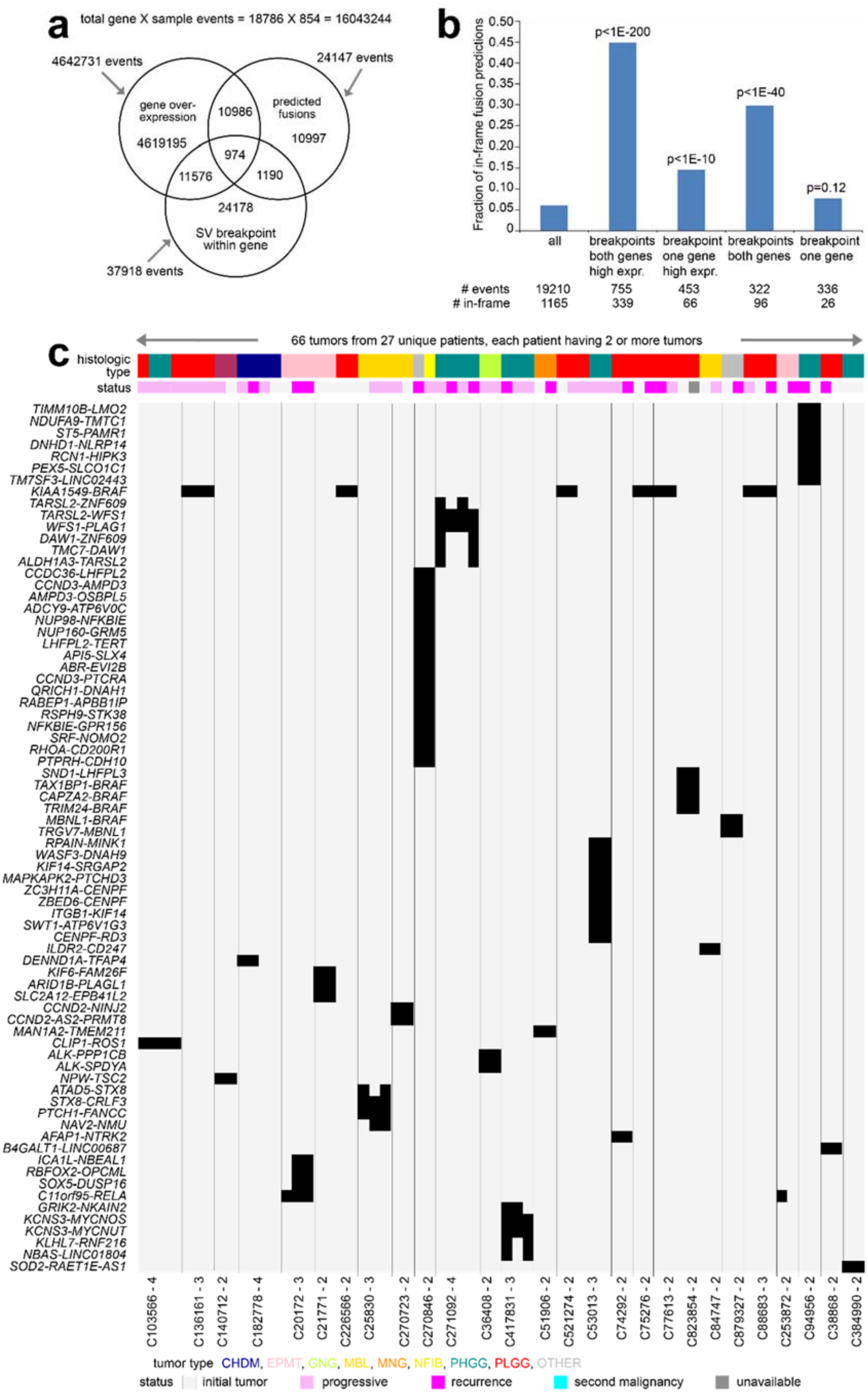
a
total gene X sample events = 18786 X 854 = 16043244
4642731 events
24147 events
gene over-expression
predicted fusions
10986
4619195
974
10997
11576
1190
24178
SV breakpoint within gene
37918 events
b
Fraction of in-frame fusion predictions
0.50
0.45
0.40
0.35
0.30
0.25
0.20
0.15
0.10
0.05
0
p<1E-200
p<1E-10
p<1E-40
p=0.12
all
breakpoints both genes high expr.
breakpoint one gene high expr.
breakpoints both genes
breakpoint one gene
events 19210 755 453 322 336
in-frame 1165 339 66 96 26
c
66 tumors from 27 unique patients, each patient having 2 or more tumors
histologic type
status
TIMM10B-LMO2
NDUFA9-TMTC1
ST5-PAMR1
DNHD1-NLRP14
RCN1-HIPK3
PEX5-SLCO1C1
TM7SF3-LINC02443
KIAA1549-BRAF
TARSL2-ZNF609
TARSL2-WFS1
WFS1-PLAG1
DAW1-ZNF609
TMC7-DAW1
ALDH1A3-TARSL2
CCDC36-LHFPL2
CCND3-AMPD3
AMPD3-OSBPL5
ADCY9-ATP6V0C
NUP98-NFKBIE
NUP160-GRM5
LHFPL2-TERT
API5-SLX4
ABR-EVI2B
CCND3-PTCRA
QRICH1-DNAH1
RABEP1-APBB1IP
RSPH9-STK38
NFKBIE-GPR156
SRF-NOMO2
RHOA-CD200R1
PTPRH-CDH10
SND1-LHFPL3
TAX1BP1-BRAF
CAPZA2-BRAF
TRIM24-BRAF
MBNL1-BRAF
TRGV7-MBNL1
RPAIN-MINK1
WASF3-DNAH9
KIF14-SRGAP2
MAPKAPK2-PTCHD3
ZC3H11A-CENPF
ZBED6-CENPF
ITGB1-KIF14
SWT1-ATP6V1G3
CENPF-RD3
ILDR2-CD247
DENND1A-TFAP4
KIF6-FAM26F
ARID1B-PLAGL1
SLC2A12-EPB41L2
CCND2-NINJ2
CCND2-AS2-PRMT8
MAN1A2-TMEM211
CLIP1-ROS1
ALK-PPP1CB
ALK-SPDYA
NPW-TSC2
ATAD5-STX8
STX8-CRLF3
PTCH1-FANCC
NAV2-NMU
AFAP1-NTRK2
B4GALT1-LINC00687
ICA1L-NBEAL1
RBFOX2-OPCML
SOX5-DUSP16
C11orf95-RELA
GRIK2-NKAIN2
KCNS3-MYCNOS
KCNS3-MYCNUT
KLHL7-RNF216
NBAS-LINC01804
SOD2-RAET1E-AS1
C103566 - 4
C136161 - 3
C140712 - 2
C182778 - 4
C20172 - 3
C21771 - 2
C226566 - 2
C25830 - 3
C270723 - 2
C270846 - 2
C271092 - 4
C36408 - 2
C417831 - 3
C51906 - 2
C521274 - 2
C53013 - 3
C74292 - 2
C75276 - 2
C77613 - 2
C823854 - 2
C84747 - 2
C879327 - 2
C88683 - 3
C253872 - 2
C94956 - 2
C38868 - 2
C384990 - 2
tumor type CHDM, EPMT, GNG, MBL, MNG, NFIB, PHGG, PLGG, OTHER
status initial tumor progressive recurrence second malignancy unavailable

Supplementary Figure 4, related to Figure 4. Additional information regarding gene fusion events identified by both RNA-seq and SSVs. (a) Considering all [gene X tumor] associations considered in this study (18786 genes X 854 CBTTC tumors, based on the set of genes represented in the RNA-seq expression dataset), Venn diagram represents the intersections involving the set of gene overexpression events (gene expression within the given tumor sample >0.4SD from the median across tumors), the set of predicted fusions by RNA-seq (using Arriba or STAR-fusion algorithms), and the set of events involving an SSV breakpoint falling within the boundary of a gene. All the overlaps represented in this diagram have a significance of p~0 by chi-squared test. Only fusions for which both genes were included in the RNA-seq dataset are represented here. **(b)** Fraction of in-frame fusion predictions by Arriba or STAR-fusion algorithms, for all candidate fusion events, and for fusion events with SSV breakpoints being found within one or both genes, with or without a high expression association, as indicated (same fusion event categories as represented in main Figure 4a, see Methods). A fusion product is in-frame when there is no frame shift in the 3′-gene, regardless whether there is single amino acid mutation or single/multiple amino acid insertion at the fusion junction point[2]. P-values by one-sided Fisher's exact test. **(c)** Fusion events involving multiple tumors from the same patient. Taking a set of gene fusions with both RNA-seq and SSV support and involving high expression (Figure 4), the subset of fusions involving two or more tumors, of the subset of tumors for which multiple tumors were profiled from the same patient, are represented here. In total, 74 different fusions across 66 tumors from 27 patients for which multiple tumors were profiled are represented. A number of these fusion events appear "ubiquitous" for a given patient, suggesting that these events might occur earlier over the course of the disease process[3].

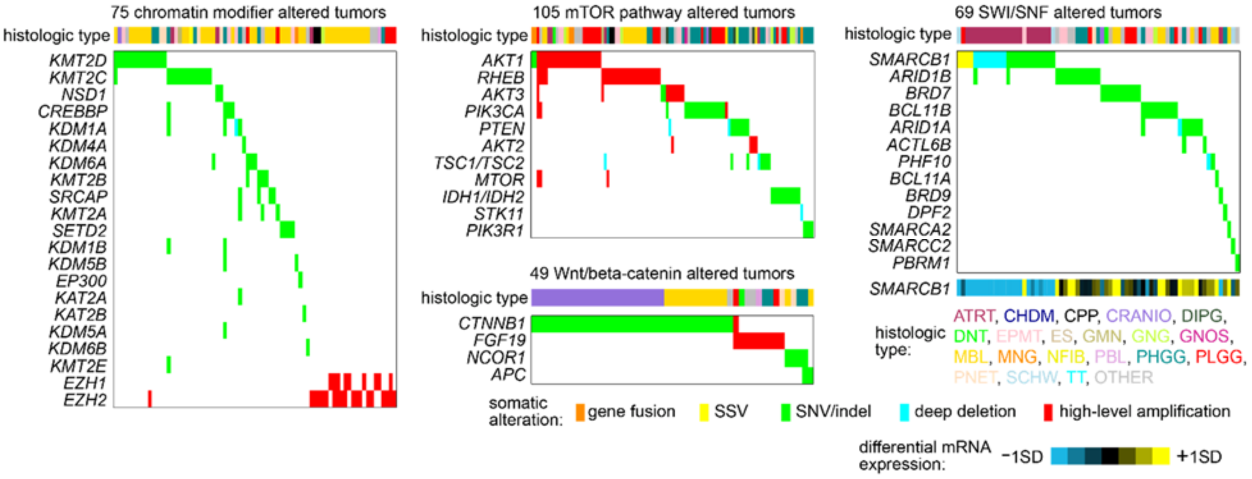

Supplementary Figure 5, related to Figure 5. Additional information regarding key oncogenic or tumor-suppressive pathways. For the pathways from Figure 5b that involved fewer than 5 SSV events (i.e. that pathways represented in Figure 5b but not in Figure 5c), somatic alteration events involving each gene included in the pathway are represented. Events are colored according to the type of somatic alteration: SNV or indel (for oncogenes, SNV within hotspot residue[4]; for tumor suppressor genes, SNV within hotspot residue or inactivating mutation by indel/nonsense/nonstop), and deep deletion or high-level amplification (respectively approximating total copy loss and copy levels more than 2X greater than that of wild-type, based on thresholded values). *PTCH1* truncation mutations (not shown) were also found in medulloblastoma in CBTTC cohort, involving 11 tumors and eight patients. One medulloblastoma tumor had an *SUFU* mutation.

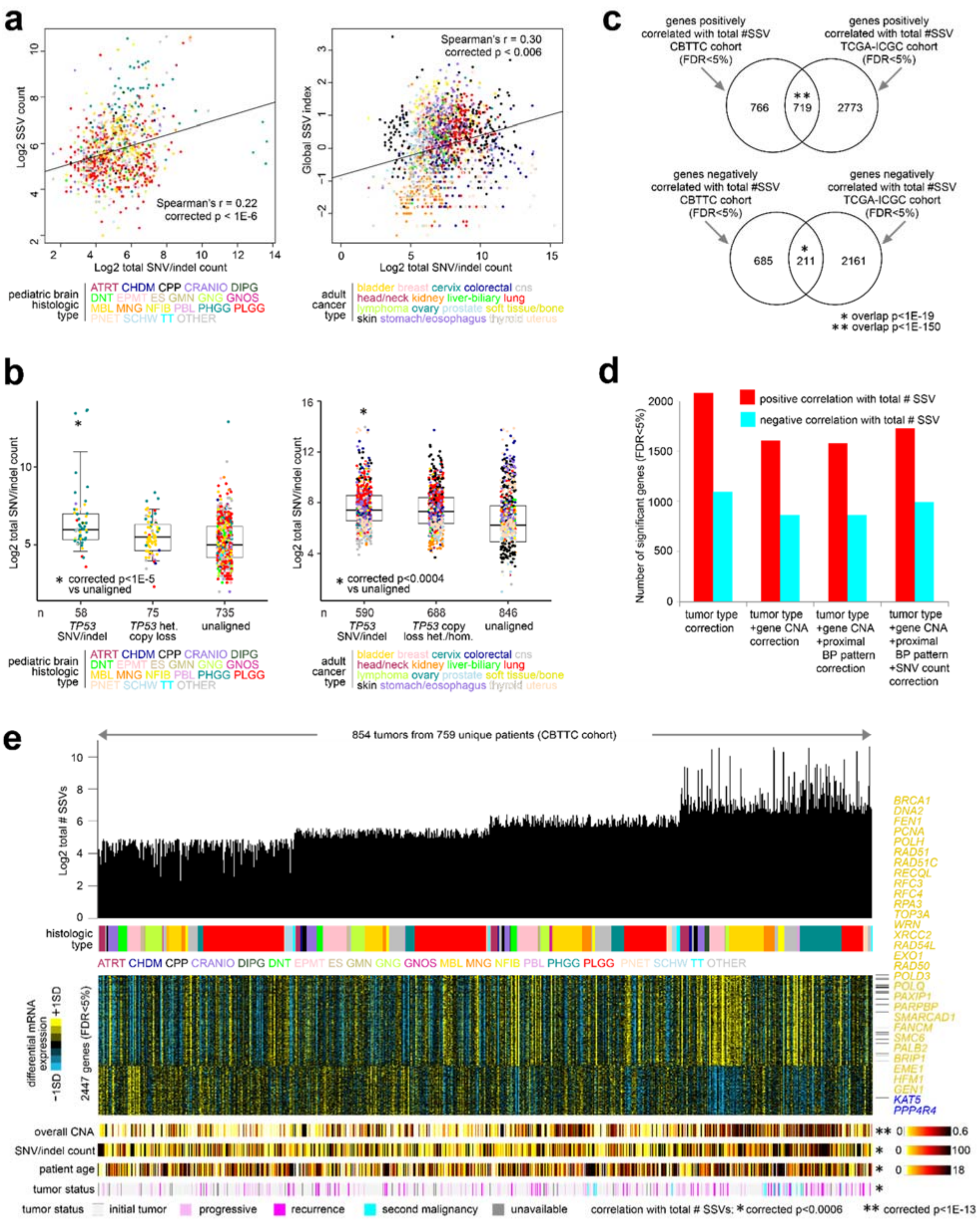

Supplementary Figure 6, related to Figure 7. Additional information regarding molecular alterations associated with the overall burden of structural variation across pediatric

brain tumors. (a) For both CBTTC cohort (left) and TCGA adult pan-cancer cohort (right), scatter plot of the total SNV/indel exome mutation count versus the total number of SSVs detected. P-values by linear model correcting for tumor type. **(b)** For both CBTTC cohort (left) and TCGA adult pan-cancer cohort (right), association of TP53 mutation or copy loss with overall SNV/indel count (based on exome analysis). P-values by linear model correcting for tumor type. Box plots represent 5% (lower whisker), 25% (lower box), 50% (median), 75% (upper box), and 95% (upper whisker). **(c)** Venn diagrams representing the overlaps between the genes positively correlated (FDR<5%, correcting for tumor type and gene-level CNA) with increasing SSVs in CTBBC pediatric brain cohort and the genes positively correlated (FDR<5%) with SSV breakpoint in either TCGA-ICGC cohort (top, n=2334 cases) or TCGA glioma cohort (bottom, n=107 GBM/LGG cases). P-values by one-sided Fisher's exact test. **(d)** Numbers of significant genes (FDR < 5%), showing correlation between expression and the total number of SSV events detected across the 107 TCGA glioma cases. Linear regression models evaluated significant associations when correcting for specific covariates, as indicated. SNV count based on exome analysis. **(e)** Across the 854 CBTTC tumors, with tumors ranked by the total number of SSVs detected, selected molecular features are represented, including top expression correlates with the total number of SSV events (from Figure 6d, tumor type+gene CNA model), overall CNA, SNV/indel mutation count (based on exome analysis), and patient age. Expression values are normalized or centered within each tumor type. Highlighted genes are homologous recombination genes as cataloged in ref[5]. P-values for correlation with the total number of SSVs by linear model correcting for tumor type.

a

gene	# samples with exome mutation: Missense Mutation	Indel	Splice Site	Nonsense Mutation
H3F3A	36	0	0	0
H3F3B	1	0	3	0
H3F3C	9	7	0	0
HIST1H3A	0	0	0	0
HIST1H3B	0	0	0	0
HIST1H3C	0	0	0	0
HIST1H3D	0	0	0	0
HIST1H3E	0	0	0	0
HIST1H3F	0	0	0	0
HIST1H3G	0	0	0	0
HIST1H3H	0	0	0	0
HIST1H3I	0	0	0	0
HIST1H3J	1	0	0	0
HIST2H3A	0	0	0	0
HIST2H3C	0	0	0	0
HIST2H3D	3	0	0	0
HIST3H3	3	0	0	0

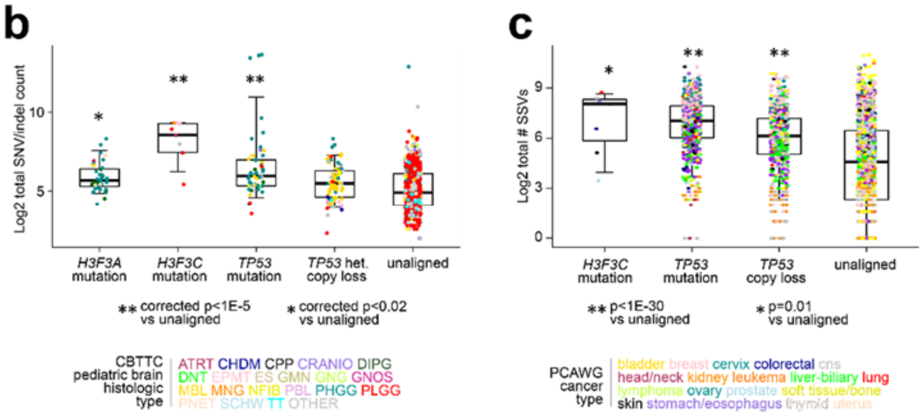

Supplementary Figure 7, related to Figure 8. Additional information regarding histone H3.3 somatic alterations in pediatric brain tumors. (a) For a set of genes encoding a member of the histone H3 family, number of CBTTC tumors for which a mutation by exome analysis was found. Of these genes, *H3F3A* and *H3F3C* appear significant, as in the case of *H3F3A* the missense mutations are highly recurrent and in the case of *H3F3C* both missense mutations and insertions are recurrent. **(b)** For CBTTC cohort, associations of somatic alterations involving *H3F3A*, *H3F3C*, or *TP53* with total number of detected SNV/indel mutations (based on exome analysis). Tumors in each group: 36, 9, 58, 75, and 711, respectively. P-values by linear model correcting for tumor type. **(c)** For PCAWG cohort, associations of somatic alterations involving *H3F3C* or *TP53* with total number of detected SSVs. Tumors in each group: 7, 668, 621, and 1500, respectively P-values by t-test using log-transformed data. Box plots represent 5% (lower whisker), 25% (lower box), 50% (median), 75% (upper box), and 95% (upper whisker).

References

1. Storey, J.D. & Tibshirani, R. Statistical significance for genomewide studies. *Proc Natl Acad Sci USA* **100**, 9440-9445 (2003).
2. Asmann, Y.*, et al.* A novel bioinformatics pipeline for identification and characterization of fusion transcripts in breast cancer and normal cell lines. *Nucleic Acids Res* **39**, e100 (2011).
3. Gerlinger, M.*, et al.* Intratumor heterogeneity and branched evolution revealed by multiregion sequencing. *N Engl J Med* **366**, 883-892 (2012).
4. Chang, M.*, et al.* Identifying recurrent mutations in cancer reveals widespread lineage diversity and mutational specificity. *Nat Biotechnol* **34**, 155-163 (2016).
5. Knijnenburg, T.*, et al.* Genomic and Molecular Landscape of DNA Damage Repair Deficiency across The Cancer Genome Atlas. *Cell Rep* **23**, 239-254.e236 (2018).
