# Supplementary material for: A pediatric brain tumor atlas of genes deregulated by somatic genomic rearrangement
Source: Nat Commun. 2021 Feb 10;12:937. doi: 10.1038/s41467-021-21081-y (PMC7876141; doi:10.1038/s41467-021-21081-y)
Supplement: Supplementary file 3 — Description of Additional Supplementary Files [file 41467_2021_21081_MOESM3_ESM.pdf]

Description of Additional Supplementary Files

File Name: Supplementary Data 1
Description: CBTTC cancer cases examined in this study (854 tumors with both WGS and RNA-seq data). Provided as an Excel file.

File Name: Supplementary Data 2
Description: Complete set of gene-level correlations between expression and nearby SSV breakpoint in CTBBC cohort, according to region examined (e.g., 0-100kb upstream, 0-100kb downstream, within the gene body, or 1Mb upstream or downstream) and the regression model applied (with or without CNA correction). For the 1Mb region window, relative gene distances of the breakpoints are weighted in the model. A tab with the top genes with FDR<10% (1Mb region window, correcting for CNA, with FDR by Story and Tibshirani method) is provided, with corresponding gene-level FDR estimations by permutation testing also being provided (see Supplementary Figure 2a). A separate tab provides the SSVs associated with enhancer hijacking and gene over-expression. Provided as an Excel file.

File Name: Supplementary Data 3
Description: Complete set of gene-level correlations between expression and nearby SSV breakpoint for each individual tumor type (using regression models with corrections for tumor type and CNA, with 1Mb region upstream or downstream of gene using weighted relative distance). In all, 13 pediatric brain tumor types were analyzed separately: ATRT, CPP, CRANIO, DNT, EPMT, GNG, MBL, MNG, NFIB, PHGG, PLGG, PNET, and SCHW. P-values by linear model. Provided as an Excel file.

File Name: Supplementary Data 4
Description: Gene fusion predictions made by RNA-seq which also have support from WGS analysis in CBTTC cohort. Provided as an Excel file.

File Name: Supplementary Data 5
Description: Pathway-level genetic and genomic alterations across CBTTC cohort, along with SSV-associated alterations. For selected pre-defined pathways involving SSV-altered genes, associated somatic SNVs, indels, CNA events, gene fusions, and SSV breakpoint events were catalogued across 854 CBTTC cancers (using cases with available RNA-seq and WGS data). Provided as an Excel file.

File Name: Supplementary Data 6
Description: Complete set of gene-level correlations between expression and nearby SSV breakpoint in CTBBC cohort (1Mb upstream or downstream), as applied separately to the subset of initial tumors (n=633) and to the subset of recurrent or progressive tumors (n=174). For the 1Mb region window, relative gene distances of the breakpoints are weighted in the model. P-values by linear model. Provided as an Excel file.

File Name: Supplementary Data 7
Description: Complete set of molecular-level correlations with the total number of SSV events detected across tumors, according to the regression model applied (i.e., what covariates were considered, all models including tumor type). Also includes Gene Ontology (GO) terms associated with genes positively correlated (FDR<5%, linear model with corrections for tumor type and CNA) with the total number of SSV events. Provided as an Excel file.
