# Supplementary material for: A pediatric brain tumor atlas of genes deregulated by somatic genomic rearrangement
Source: Nat Commun. 2021 Feb 10;12:937. doi: 10.1038/s41467-021-21081-y (PMC7876141; doi:10.1038/s41467-021-21081-y)
Supplement: Supplementary file 11 — Reporting Summary [file 41467_2021_21081_MOESM11_ESM.pdf]

nature research

Corresponding author(s): Chad Creighton

Last updated by author(s): Nov 10, 2020

Reporting Summary

Nature Research wishes to improve the reproducibility of the work that we publish. This form provides structure for consistency and transparency in reporting. For further information on Nature Research policies, see Authors & Referees and the Editorial Policy Checklist .

Statistics

For all statistical analyses, confirm that the following items are present in the figure legend, table legend, main text, or Methods section.

n/a	Confirmed	
☐	☒	The exact sample size (*n*) for each experimental group/condition, given as a discrete number and unit of measurement
☐	☒	A statement on whether measurements were taken from distinct samples or whether the same sample was measured repeatedly
☐	☒	The statistical test(s) used AND whether they are one- or two-sided<br>*Only common tests should be described solely by name; describe more complex techniques in the Methods section.*
☐	☒	A description of all covariates tested
☐	☒	A description of any assumptions or corrections, such as tests of normality and adjustment for multiple comparisons
☐	☒	A full description of the statistical parameters including central tendency (e.g. means) or other basic estimates (e.g. regression coefficient) AND variation (e.g. standard deviation) or associated estimates of uncertainty (e.g. confidence intervals)
☐	☒	For null hypothesis testing, the test statistic (e.g. *F*, *t*, *r*) with confidence intervals, effect sizes, degrees of freedom and *P* value noted<br>*Give P values as exact values whenever suitable.*
☐	☒	For Bayesian analysis, information on the choice of priors and Markov chain Monte Carlo settings
☐	☒	For hierarchical and complex designs, identification of the appropriate level for tests and full reporting of outcomes
☐	☒	Estimates of effect sizes (e.g. Cohen's *d*, Pearson's *r*), indicating how they were calculated

Our web collection on statistics for biologists contains articles on many of the points above.

Software and code

Policy information about availability of computer code

Data collection	All data used in this study are publicly available. Source code in R for the linear modeling integrating SSV with expression data, with example data files, is available at Github https://github.com/chadcreighton/SVexpression_integration.
Data analysis	Analysis in R using version 4.0.3.

For manuscripts utilizing custom algorithms or software that are central to the research but not yet described in published literature, software must be made available to editors/reviewers. We strongly encourage code deposition in a community repository (e.g. GitHub). See the Nature Research guidelines for submitting code & software for further information.

Data

Policy information about availability of data

All manuscripts must include a data availability statement. This statement should provide the following information, where applicable:

- Accession codes, unique identifiers, or web links for publicly available datasets
- A list of figures that have associated raw data
- A description of any restrictions on data availability

We accessed the SSV VCF files through the public project on the Kids First Data Resource Portal and Cavatica at https://cavatica.sbgenomics.com/u/cavatica/pbta-cbttc/files/#q?path=structural-variations. Gene-level copy number alteration (CNA) calls, made based on CBTTC WGS data, were obtained from the PedCBioPortal (https://pedcbioportal.org/datasets). We obtained processed RNA-seq data for CBTTC samples from the PedCBioPortal (https://pedcbioportal.org/).

Field-specific reporting

Please select the one below that is the best fit for your research. If you are not sure, read the appropriate sections before making your selection.

Life sciences study design

All studies must disclose on these points even when the disclosure is negative.

Sample size	The set of samples with both RNA-seq and WGS-based SSV calls, as made available from CBTTC data repositories on September 2019, were used in the study.
Data exclusions	The set of samples with both RNA-seq and WGS-based SSV calls were used in the study. No samples meeting the above criteria were excluded.
Replication	Not applicable, no experimental data.
Randomization	Not applicable, no experimental data.
Blinding	Blinding not relevant, as samples were not allocated to experimental groups.

Reporting for specific materials, systems and methods

We require information from authors about some types of materials, experimental systems and methods used in many studies. Here, indicate whether each material, system or method listed is relevant to your study. If you are not sure if a list item applies to your research, read the appropriate section before selecting a response.

Materials & experimental systems

n/a	Involved in the study	
☒	☐	Antibodies
☒	☐	Eukaryotic cell lines
☒	☐	Palaeontology
☒	☐	Animals and other organisms
☒	☐	Human research participants
☒	☐	Clinical data

Methods

n/a	Involved in the study	
☒	☐	ChIP-seq
☒	☐	Flow cytometry
☒	☐	MRI-based neuroimaging
